# Treatment of patients with carcinomas in advanced stages with 5-fluorouracil, folinic acid and pyridoxine in tandem

**DOI:** 10.1038/s41598-024-62860-z

**Published:** 2024-05-27

**Authors:** David Machover, Wathek Almohamad, Vincent Castagné, Christophe Desterke, Léa Gomez, Emma Goldschmidt

**Affiliations:** 1 INSERM U935-UA09, University Paris-Saclay, Paul-Brousse Hospital, 12, Avenue Paul-Vaillant-Couturier, 94800 Villejuif, France; 2 Department of Medical Oncology, University Paris-Saclay, Paul-Brousse Hospital, Assistance Publique-Hôpitaux de Paris (APHP), 94800 Villejuif, France; 3 Department of Pharmacy, University Paris-Saclay, Paul-Brousse Hospital, APHP, 94800 Villejuif, France; 4 Department of Biophysics and Nuclear Medicine, University Paris-Saclay, Kremlin-Bicêtre Hospital, APHP, 94270 Le Kremlin-Bicêtre, France

**Keywords:** Cancer, Chemotherapy, Drug development

## Abstract

The effect of high-dose pyridoxine (PN) on activity of 5-fluorouracil (FUra) and folinic acid (FA)-containing regimens was studied in 50 patients including 14 with digestive tract, and 36 with breast carcinomas (BC) in advanced stages with poor prognostic characteristics. Patients with colorectal, and pancreas adenocarcinoma received oxaliplatin, irinotecan, FUra, FA (*Folfirinox*), and patients with squamous cell carcinoma of the esophagus had paclitaxel, carboplatin, FUra, FA (*TCbF*). Patients with BC received *AVCF* (doxorubicin, vinorelbine, cyclophosphamide, FUra, FA) followed by *TCbF* or *TCbF* only*,* and patients who overexpressed HER2 received *TCbF* plus trastuzumab and pertuzumab. PN (1000–3000 mg/day iv) preceded each administration of FUra and FA. 47 patients (94%) responded, including 16 (32%) with CR. Median tumor reduction was 93%. Median event-free survival (EFS) was 37.7 months. The 25 patients with tumor shrinkage ≥ 91% had EFS of 52% from 42 months onwards. Unexpected toxicity did not occur. PN enhances potency of chemotherapy regimens comprising FUra and FA.

## Introduction

**Figure 1:**
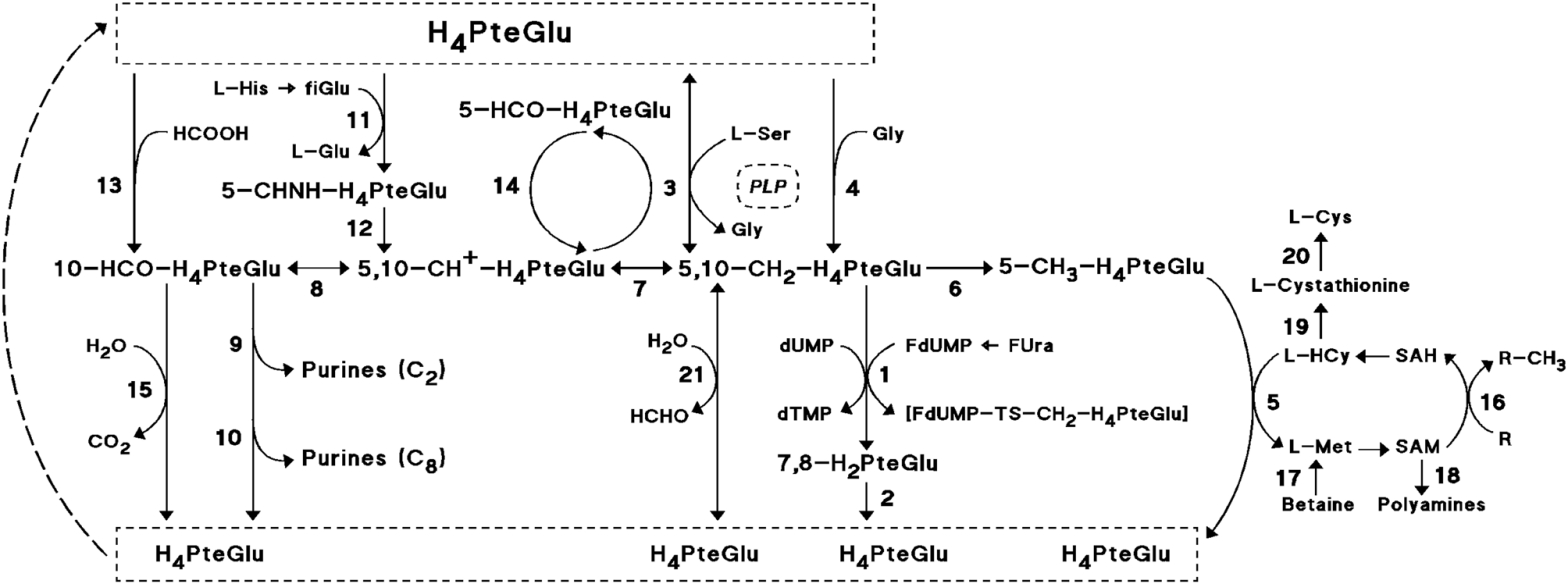
Selected Pathways of Folates. The ternary complex [FdUMP-TS-CH_2_-H_4_PteGlu]. **Folates.** 7,8-H_2_PteGlu: 7,8-dihydrofolate; H_4_PteGlu: [6S]-tetrahydrofolate; 5,10-CH_2_-H_4_PteGlu: [6R]-5,10-methylenetetrahydrofolate; 5-CH_3_-H_4_PteGlu: [6S]-5-methyltetrahydrofolate; 5,10-CH^+^-H_4_PteGlu: [6R]-5,10-methenyltetrahydrofolate; 10-HCO-H_4_PteGlu: [6R]-10-formyltetrahydrofolate; 5-CHNH-H_4_PteGlu: [6S]-5-formiminotetrahydrofolate; 5-HCO-H_4_PteGlu: [6S]-5-formyltetrahydrofolate (folinic acid; leucovorin). **Enzymes and non-enzymatic chemical reactions.** (1) Thymidylate synthase (TS); (2) dihydrofolate reductase; (3) pyridoxal 5′-phosphate (PLP)-dependent serine hydroxymethyltransferase (including the cytoplasmic SHMT1 and the mitochondrial SHMT2 isoforms); (4) glycine cleavage system (including the carrier H protein bound to the mitochondrial inner membrane, and the enzymes P protein, a PLP-dependent glycine dehydrogenase, T protein, an amino methyltransferase, and L protein, a dihydro lipoamide dehydrogenase); (5) methionine synthase; (6) methylenetetrahydrofolate reductase; (7) methylenetetrahydrofolate dehydrogenase; (8) methenyltetrahydrofolate cyclohydrolase; (9) Phosphoribosylglycinamide formyltransferase; (10) aminoimidazole carboxamide ribonucleotide formyltransferase; (11) formiminotransferase cyclodeaminase; (12) formiminotetrahydrofolate cyclodeaminase; (13) formate tetrahydrofolate ligase; (14) methenyl tetrahydrofolate synthetase/5-formyltetrahydrofolate cycloligase; (15) 10-formyltetrahydrofolate dehydrogenase; (16) activated methyl transfer enzymes; (17) betaine-homocysteine methyltransferase; (18) adenosylmethionine decarboxylase; the PLP-dependent (19) cystathionine β-synthase, and (20) cystathionine γ-lyase; (21) non-enzymatic reaction between 5,10-methylenetetrahydrofolate, formaldehyde, and tetrahydrofolate. Arrows in circle represent the 5-HCO-H_4_PteGlu : 5,10-CH^+^-H_4_PteGlu futile cycle. **Metabolites, and compounds.** dUMP, deoxyuridylate; dTMP, thymidylate; SAH, S-adenosyl-L-homocysteine; SAM, S-adenosyl-L-methionine; L-HCy, L-homocysteine; L-Met, L-methionine; L-Cys, L-cysteine; L-His, L-histidine; fiGlu, formiminoglutamic acid; FUra, 5-fluorouracil; FdUMP, fluoro deoxyuridylate; [FdUMP-TS-CH_2_-H_4_PteGlu], the ternary complex; PLP, pyridoxal 5′-phosphate; HCHO, formaldehyde; HCOOH, formic acid.

The fluoropyrimidine 5-fluorouracil (FUra) exerts powerful antitumor effect in cancer cells resulting from inhibition of thymidylate synthase (TS) and, to an undetermined degree, from incorporation into RNA that disrupts RNA processing and function^1^. Fluorodeoxyuridine monophosphate (FdUMP), the metabolite of FUra-mediated inhibition of TS, prevents synthesis of thymidine triphosphate (dTTP) leading to deoxynucleotide triphosphate (dNTP) pool imbalance together with deoxyuridine triphosphate (dUTP) and fluorodeoxyuridine triphosphate (FdUTP) build-up, which results in genomic DNA replication defects including DNA mismatch and altered replication fork progression eliciting DNA damage cell responses through several distinct signaling pathways and, ultimately, cell death^1–5^. FdUMP binds to TS and the folate cofactor [6R]-5,10-methylene tetra hydro pteroylglutamate (CH_2_-H_4_PteGlu) to form a TS-inactivating covalently bound ternary complex [TS-FdUMP-CH_2_-H_4_PteGlu], whose dissociation decreases as CH_2_-H_4_PteGlu is augmented up to concentrations greater than 1 mM (Fig. 1)^6–8^. Supplementation of cancer cells exposed to FUra or fluorodeoxyuridine (FdUrd) with high concentration 5-formyl tetra hydro pteroylglutamate [5-HCO-H_4_PteGlu; folinic acid (FA); leucovorin] in vitro leads to greater formation of ternary complex than with the fluoropyrimidines as single agents, resulting in potentiation of the cytotoxic effect^9^. These findings led the way to regimens of FUra combined with FA possessing greater antitumor efficacy than FUra as a single agent that are used as standards for treatment of patients with digestive tract carcinomas^10–12^.

FUra is listed as one of essential medicines for treatment of several types of cancer but, despite enhancement of antitumor activity by addition of folinic acid, its importance is underrated. This may result from the incapacity of this cytostatic agent to fully deploy its effect in tumors that are not metabolically prepared under natural conditions for optimum FdUMP-mediated inhibition of the TS. To circumvent this limitation, we elaborated a new pharmacologic modulation by using high amounts of pyridoxine (PN), an unphosphorylated B6 vitamer, and folinic acid in tandem with the intent at providing cancer cells with transient metabolic changes permitting the cytotoxic effect of FUra to be improved^13^.

Augmentation of CH_2_-H_4_PteGlu pools by exposure of cancer cells to high amounts of any reduced folate occurs to a small extent. Studies of CH_2_-H_4_PteGlu concentration changes in cancer cells exposed to 5-HCO-H_4_PteGlu either in [6R,S]- or the natural [6S]-form, to 5-methyl tetra hydro pteroylglutamate (CH_3_-H_4_PteGlu), to 5,6,7,8-tetra hydro pteroylglutamate (H_4_PteGlu), or to 5,10 methylene tetra hydro pteroylglutamate ([6R]-5,10-CH_2_-H_4_PteGlu) have demonstrated that supplementation with high amounts of these folates results in limited increase of CH_2_-H_4_PteGlu concentration of variable degree up to levels far below that required to increase the tightness of FdUMP binding to TS for optimum stability of the ternary complex^7,8^, followed by rapid decline after discontinuation of folate exposure^14–23^. Poor intracellular expansion of CH_2_-H_4_PteGlu pools is a consequence of the great turnover of folates in cancer cells resulting from mechanisms leading to their rapid interconversion including the methylene tetrahydrofolate reductase (MTHFR)-catalyzed irreversible reduction of CH_2_-H_4_PteGlu to CH_3_-H_4_PteGlu, the 10-formyl tetrahydrofolate dehydrogenase-catalyzed dehydrogenation of 10-formyl tetra hydro pteroyl glutamate (10-HCO-H_4_PteGlu) to H_4_PteGlu, the non-enzymatic reaction between CH_2_-H_4_PteGlu, formaldehyde, and H_4_PteGlu, and the 5,10-methenyl tetra hydro pteroyl glutamate (5,10-CH^+^-H_4_PteGlu) : 5-HCO-H_4_PteGlu futile cycle (Fig. 1)^24–28^. Limitation of CH_2_-H_4_PteGlu expansion is also due to restricted synthesis of CH_2_-H_4_PteGlu from H_4_PteGlu, the pivotal folate, that results mostly from transfer of Cβ of L-serine to H_4_PteGlu catalyzed by serine hydroxymethyl transferase (SHMT), a ubiquitous pyridoxal 5′-phosphate (PLP)-dependent enzyme that is the major source of one-carbon units for cellular metabolism^29–32^.

The rationale for augmenting the cytotoxicity of the fluoropyrimidines by folates and vitamin B6 in tandem lies in the low affinity for binding of apo-SHMT to cofactor. PLP was found to bind mammalian SHMT including man with dissociation constant (K_d_) much higher than PLP concentrations present in cells under natural conditions^13,29–34^, which indicates that SHMT activity should be sensitive to PLP level variations. Changes of folate-mediated one-carbon metabolism and SHMT activity in relation with vitamin B6 availability were demonstrated experimentally. Vitamin B6 deficiency was accompanied by reduced SHMT activity and decreased methylation of L-homocysteine to L-methionine with methyl groups from L-serine i.e., resulting from SHMT-catalyzed synthesis of CH_2_-H_4_PteGlu, and subsequently of CH_3_-H_4_PteGlu, a cofactor of methionine synthase^31,35,36^. Experiments also found that large proportions of SHMT in cells supplied with sufficient vitamin B6 to support normal growth lie as inactive apoenzyme^31,36^.

We hypothesized that, in tumors, naturally occurring PLP levels are too small to allow SHMT-dependent conversion of H_4_PteGlu into CH_2_-H_4_PteGlu in amounts required to improve FdUMP-mediated inhibition of TS by increasing stability of the ternary complex. To assess for variations of SHMT activity resulting from PLP concentration changes, we performed experiments in human and mouse cancer cell lines to investigate for interactions between FUra, FA, and PLP on cell growth^13^. Supplementation of cells exposed to FUra with high concentration PLP and folinic acid in tandem resulted in powerful synergistic potentiation of FUra cytotoxicity. Parenteral administration of high dose pyridoxine (PN) or pyridoxamine (PM) in mice increased erythrocyte levels of PLP up to concentrations within the range of K_d_ values of SHMT binding to cofactor^13,29,31,32,37^. From these data, we thought that administration of high-dose unphosphorylated B6 vitamer to patients treated with FUra and FA would increase PLP levels in tumors, leading to augmentation of CH_2_-H_4_PteGlu synthesis resulting in TS inhibition of great magnitude and extended duration for enhanced antitumor effect.

Based on these experimental findings we conducted a pilot clinical study for treatment of patients with digestive tract, and breast carcinomas in advanced stages with poor prognostic features who were not amenable to resection, radiotherapy, nor to any chemotherapy with curative intent, and whose standard chemotherapy regimens included a combination of FUra and FA, consisting in addition of high dose pyridoxine to these regimens. Early results were previously reported^37,38^. We present herein now the final update that included fifty patients entered from June 2014 to March 2023. With higher number of patients and extended follow-up time, results are remarkably favorable in the continuity of that previously reported.

## Methods

Approval of study was granted prospectively by the board of the Medical Oncology Department in Paul-Brousse Hospital, Assistance Publique-Hôpitaux de Paris, University Paris-Saclay. Registration was not required for patients being selected as carrying poor prognostic features receiving standard regimens of chemotherapy whose components, including FUra modulators, belong to the public domain. The pilot study was conducted in accordance with the principles of the Declaration of Helsinki. All the patients were informed of the rationale, potential benefits, and risks of the treatment. Written informed consent to study participation was obtained from all patients.

## Patients

Patients with carcinomas of the digestive tract and with breast carcinomas in advanced stages carrying unfavorable prognostic features were entered in a single clinical center, including patients with poor performance status (PS) scores whose nutritional and vital function conditions were sufficiently preserved to allow chemotherapy being administered cautiously in safe conditions. Owing to the great extent of tumor and poor PS scores in many, patients could not be selected neither for resection, radiotherapy, chemotherapy with curative intent nor for any investigational therapy available (Tables 1, and 2).

**Table 1:** Characteristics of patients with advanced carcinomas of the digestive tract treated with regimens including FUra, folinic acid and pyridoxine in tandem. Results of therapy.

Type	No.	Age (Sex)	Primary [Sites of metastatic involvement and local tumor extension]	Stage (ECOG PS)	Treatment regimen^1^ (Median daily PN dose in mg × 10^3^)	Antitumor activity			Marker ratio (B/A)^4^	Time to response(mos)	EFS (mos)
						RECIST (Percent change)	PERCIST (Percent change)	Pathologic^3^			
I	1	65 (M)	Caecum. Ras WT [Peritoneum]	IV (2)	*Folfirinox*^2^ (1.5)	–100	na	na	na	1.9	42
	2	61 (M)	Sigmoid. K-Ras mut [Lung; Peritoneum]	IV (1)	*Folfirinox* (3)	–91	–100	Absent^α^	22/3^a^	4.7	17
	3	37 (F)	Right colon. K-Ras mut [Liver]	IV (4)	*Folfirinox* (3)	–87	–100	ypT3N1bM1^β^	6667/4^a^	1.4	9
	4	60 (F)	Rectum. K-Ras mut [Liver; Lung; Pleura; Bone; Nodes]	IV (4)	*Folfirinox* (3)	–86	na	na	2376/16^b^	2.7	18
	5	61 (F)	Right colon. K-Ras mut [Lung; Peritoneum; Nodes]	IV (2)	*Folfirinox* (3)	–81	–100	ypT0N0M0^β^	na	3	24
	6	49 (M)	Caecum. Ras WT [Liver; Peritoneum; Nodes]	IV (3)	*Folfirinox*^2^ (1)	–78	–69	ypT4bN2aM1^β^	2073/16^b^	3.1	9
II	7	71 (M)	Head-body [Portal vein; Coeliac artery; Duodenum]	IV (2)	*Folfirinox* (1)	–100	na	ypT3N0M0^γ^	9728/2^b^	4.9	23
	8	69 (F)	Head [Coeliac artery; Lung; Nodes]	IV (3)	*Folfirinox* (1)	–100	–100	na	9755/25^b^	4.8	31
	9	(50) M	Tail [Coeliac artery; Liver]	IV (4)	*Folfirinox* (3)	–86	na	na	9756/2646^b^	2.2	6
	10	(61) F	Head [Coeliac artery; Liver; Lung; Nodes]	IV (3)	*Folfirinox* (1)	–79	na	na	na	3.8	14
	11	(67) M	Head. [Portal vein; Coeliac artery]	IV (3)	*Folfirinox* (1.5)	–16	na	na	na	-	-
III	12	74 (F)	Upper third within field of prior radiotherapy	usT3N1M0 (1)	*TCbF* (3)	–100	–100	Absent^α^	na	1.2	30
	13	62 (M)	Upper-mid thirds	usT2N1M0 (1)	*TCbF* (2)	–100	–100	ypT0N0M0^δ^	na	1.1	94 +
	14	F (77)	Upper-mid thirds	T3N3M0 (2)	*TCbF* (1.5)	–29	–18	na	na	-	-

Patients with colorectal adenocarcinoma (I); pancreas adenocarcinoma (II); and squamous-cell carcinoma of esophagus (III). ^1^Regimens of chemotherapy administered to patients; description in text. ^2^Cetuximab was combined to chemotherapy.^3^Methods employed for pathologic assessment were: ^α^us-guided biopsy; ^β^colectomy and resection of metastases; ^γ^duodenopancreatectomy; ^δ^esophagectomy. Results obtained for patients who were assessed by biopsy are indicated as tumor present or absent. ^4^Ratio of tumor marker with the highest initial value found in each patient (*i.e.*, ^a^CEA, or ^b^CA19-9) measured before (B) and after (A) induction therapy. Na, not applicable.

**Table 2:** Characteristics of patients with advanced breast carcinoma treated with regimens including FUra, folinic acid and pyridoxine in tandem. Results of therapy.

Type	No	Age	Primary [Sites of metastatic involvement]	Stage (ECOG PS)	Treatment regimen^1^ (Median daily PN dose in mg × 10^3^)	Antitumor activity			CA15-3 ratio (B/A)^2^	Time to response (mos)	EFS (mos)
						RECIST (percent change)	PERCIST (percent change)	Pathologic^3^			
I	1	47	T4dN3c LC EEII Ki67:10 ER + PR + [Nodes; Bone; Wall]	IV (1)	*AVCF-TCbF-VCbF* (3)	–100	–100	na	166/24	4	23
	2	50	T3N2 DC EEII Ki67:30 ER + PR + [Bone]	IV (1)	*FAC-TCbF-VCbF* (1)	–100	–100	na	na	4.5	67 +
	3	63	T4dN1 DC EEIII Ki67:70 ER + PR-	IIIB (0)	*AVCF-TCbF-VCbF* (1)	–100	–100	ypT0N0^α^	na	4.6	67 +
	4	54	T2N3b DC EEI Ki67 :15 ER + PR + [Nodes; Adrenals; Bone]	IV (1)	*TCbF-VCbF* (3)	–100	–100	na	na	3.2	28 +
	5	34	T4dN3c DC EEIII Ki67:60 ER + PR +	IIIC (0)	*AVCF-TCbF* (3)	–100	–100	ypT0N1(mi)^α^	na	2,3	14 +
	6	49	T3N3c DC EEII Ki67:40 ER + PR + [Nodes]	IV (2)	*FAC-TCbF-VCbF* (3)	–98	–100	Absent^β^	272/29	2.3	43 +
	7	53	T2N3c LC ER + PR + [Nodes; Peritoneum; Spleen; Bone]	IV (4)	*VCbF-TCbF* (2)	–98	–100	Present^γ^	2212/123	4.7	22
	8	37	T3N2 DC EEIII Ki67:35 ER + PR + [Bone]	IV (2)	*AVCF-TCbF-VCbF* (3)	–96	–100	ypT1bN0^α^	na	3.3	39 +
	9	44	T4cN2 DC EEII Ki67:22 ER + PR + [Nodes; Bone; Lung; Pleura]	IV (3)	*AVCF-TCbF-VCbF* (3)	–96	–93	na	1017/19	2.4	31 +
	10	48	T4dN3 DC EEII Ki67:30 ER + PR + [Nodes; Wall]	IV (3)	*AVCF-TCbF-VCbF* (2)	–89	–87	Present^β^	849/24	4.4	51
	11^4^	62	DC EEIII Ki67:35; ER + PR + [Nodes; Skin; Wall]	IV (0)	*TCbF-VCbF* (2.5)	–89	–84	ypN1aM0^δ^	na	3.4	55 +
	12	55	T4bN2a DC Ki67:20 ER + PR +	IIIC (1)	*AVCF-TCbF* (3)	–89	–83	na	na	3.2	9 +
	13	62	T4dN3a DC EEIII Ki67:50 ER + PR + [Lung]	IV (2)	*AVCF-TCbF-VCbF* (3)	–82	–83	ypT1bN1a^α^	na	0.5	23 +
	14	41	T4dN3a DC EEII Ki67:40 ER + PR- [Bone]	IV (2)	*AVCF-TCbF* (1)	–79	–85	ypT1cN1a^α^	na	5.3	38
	15	33	T4dN3b DC EEII Ki67:22 ER + PR +	IIIC (0)	*AVCF-TCbF* (3)	–65	–85	ypT3N1a^α^	na	3.3	25 +
	16^4^	43	DC EEIII Ki67:10 ER + PR- [Skin]	IV (0)	*TCbF-VCbF* (1)	–64	–50	ypN0M1^δ^	na	3.9	77 +
	17	43	T4dN3c DC EEIII Ki67:60 ER + PR- [Liver; Lung; Nodes; Pleura; Wall]	IV (1)	*TCbF* (1)	–62	na	na	782/73	2.5	7
	18	57	T4dN3c DC EEIII Ki67:70 ER- PR- [Lung; Node; Bone]	IV (2)	*AVCF-TCbF* (3)	–46	–78	na	na	2.4	7
II	19	52	T4dN3b DC EEII Ki67:25 ER + PR- [Nodes]	IV (1)	*TCbF-VCbF* (1)	–100	–100	ypT0N0^α^	62/25	3.9	66 +
	20	47	T4dN2 DC EEIII Ki67:30 ER- PR-	IIIB (1)	*TCbF-VCbF* (2)	–100	–100	ypT0N0^α^	na	1.7	93 +
	21	55	T2N2 DC EEII Ki67:40 ER + PR- [Liver; Lung; Bone]	IV (4)	*TCbF-VCbF* (3)	–100	–100	ypT1bNx^α^	14,750/29	2.4	27
	22	62	T4dN3b DC EEIII Ki67:70 ER- PR-	IIIC (3)	*TCbF-VCbF* (2)	–100	–93	ypT0N0^α^	na	4.2	89 +
	23	51	T4dN3c DC EEI Ki67:80 ER- PR- [Nodes; Wall]	IV (3)	*TCbF-VCbF* (1)	–98	–100	Absent^β^	na	8.4	92 +
	24	68	T2N3c DC Ki67:20 ER + PR- [Liver; Nodes; Bone; Cranial nerves]	IV (4)	*TCbF-VCbF* (3)	–98	na	na	422/26	2	45 +
	25	38	T4dN2 DC EEII Ki67:30 ER + PR +	IIIB (0)	*TCbF-VCbF* (3)	–88	–88	ypT1aN1(mi)^α^	70/25	3.2	19 +
III^5^	26^4^	75	DC ER + PR + [Bone; Cranial nerves]	IV (3)	*TCbF* (2)	na	–100	na	152/79	2.1	54
	27^4^	47	LC EEII ER + PR + [Liver; Nodes]	IV (2)	*TCbF* (1)	–100	–100	na	1272/12	2,8	13
	28^4^	68	DC EEII ER + PR- [Nodes]	IV (1)	*VCbF* (2)	–100	–93	na	116/29	2.4	34
	29^4^	45	DC EE II Ki67:70 ER + PR-	IV (1)	*TCbF* (3)	–94	–47	na	126/46	6.8	12
	30^4^	40	DC EEII Ki67:90 ER- PR- [Liver; Nodes; Bone; Wall]	IV (1)	*VCbF* (1)	–91	–100	na	72/13	2.6	50 +
	31^4^	42	DC EEII Ki67:25 ER + PR + [Lung; Bone]	IV (3)	*TCbF* (2)	–88	–100	Absent^γ^	101/14	5.4	30^6^
	32^4^	54	DC EEIII Ki67:80 ER- PR- [Liver; Nodes; Spinal cord epiduritis]	IV (4)	*TCbF* (1)	–88	–100	na	na	3.1	15
	33^4^	59	LC EEII Ki67:15 ER + PR + [Nodes; Bone; Wall]	IV (1)	*TCbF-VCbF* (3)	–86	–82	na	79/23	2.6	7 +
	34^4^	56	DC EEII Ki67:70 ER + PR- [Liver; Lung; Nodes]	IV (1)	*TCbF* (2)	–81	–91	na	na	3.3	27
	35	61	T3N3b DC ER + PR + [Liver; Lung; Nodes; Bone]	IV (2)	*AVCF-VCbF* (3)	–41	–53	na	142/72	1.6	9
	36^4^	54	DC EEIII Ki67:60 ER- PR- [Nodes; Skin]	IV (2)	*TCbF* (1)	45	na	na	na	-	-

Patients with breast carcinoma who did not receive prior chemotherapy whose tumor did not overexpress HER2 (I); patients who did not receive prior chemotherapy whose tumor overexpressed HER2 (II); and patients who received prior chemotherapy whose tumor did not overexpress HER2 (III). ^1^Regimens of chemotherapy administered to patients in succession; description in text. Patients with HER2 overexpression received chemotherapy combined with trastuzumab, and pertuzumab. ^2^Ratio of CA15-3 values measured before (B) and after (A) induction therapy. ^3^Methods used for pathologic assessment were: ^α^Mastectomy; ^β^us-guided biopsy; ^γ^bone biopsy; ^δ^resection with eradication intent of anatomic area containing residual tumor. Results obtained for patients who were assessed by biopsy are indicated as tumor present or absent. ^4^Patient who had prior mastectomy. ^5^Of eleven previously treated patients, ten have had prior anthracycline-containing chemotherapy of whom eight had taxanes as well, and one patient received paclitaxel only. Nine of eleven patients had received FUra as part of their previous regimens of chemotherapy, including five who had FUra and folinic acid. ^6^Patient died of unrelated cause without carcinoma progression. Na, not applicable.

### Patients with carcinomas of the digestive tract

Fourteen patients with three types of carcinomas of the digestive tract aged 37–77 years old (median, 61.7 years) in advanced stage who had not received prior treatment were included. Six patients aged 37–65 years old had advanced colorectal adenocarcinoma with regional invasion and numerous metastases (Table 1). Of these, two had adenocarcinoma with wild type (WT) Ras, and four patients had tumors that carried K-Ras mutations. One patient had initial resection of the primary tumor and five did not. Five patients aged 50 to 71 years old had locally advanced unresectable pancreas adenocarcinoma of which three had numerous metastases. Three patients aged 62 to 77 years old had squamous cell carcinoma of the upper and mid thirds of the esophagus that could not be resected (Table 1). Great tumor burden was measured in most patients. Eastern Cooperative Oncology Group (ECOG) PS scores at presentation were 0–1 in three patients, 2 in four patients, 3 in four patients, and 4 in three patients.

### Patients with carcinomas of the breast

Patients with ductal or lobular adenocarcinoma of the breast in advanced stages carrying poor prognostic features who did not receive prior chemotherapy as well as those who had previously received no more than two prior lines of chemotherapy were included in the study. Patients presented either with primary tumor of the breast staged T4 together with ipsilateral lymph node involvement of any extent accompanied or not with bone, nodal, skin, and/or visceral metastases; with primary and ipsilateral lymph nodes at any stage accompanied with metastases; or with metastases only (Table 2). Previously treated patients could have received one chemotherapy regimen as pre-operative or post-operative adjuvant treatment, and/or one line of treatment for advanced disease. Previous hormone therapy of any type was admitted, including hormone therapy combined with CDK 4/6 inhibitors.

Thirty-six patients aged 33–75 years old (median 51 years) were included in the study (Table 2). Of 25 patients who had not received prior chemotherapy, 7 had tumors that overexpressed (3 +) the Human Epidermal Growth Factor Receptor-2 (Her2/neu; HER2) as assessed by immunohistochemistry and 18 had tumors that did not. Of these 25 patients, sixteen had locally advanced unresectable primary accompanied with bone, nodal, soft tissue, and/or visceral metastases including 8 patients with inflammatory carcinoma (T4d); two patients had unresectable thoracic wall involvement developed in the anatomical area of prior exclusive mastectomy; and seven patients presented with unresectable primary including six patients with stage T4d, and one with stage T4b, together with ipsilateral lymph node involvement, without detectable metastases. AJCC anatomic stages were IIIB, IIIC, and IV in 3, 4, and 18 patients, respectively. Of the twenty-five previously untreated patients, twenty-one had tumors that expressed estrogen receptors (ER), and 4 had tumors that did not, including one withtriple negative carcinoma. Eleven patients with stage IV breast carcinoma diagnosed 1.5 to 25 years (mean, 8.5 years) before entering the study had received prior chemotherapy. Of these, five have had prior preoperative or adjuvant chemotherapy only, 4 had first-line chemotherapy for advanced disease only, and two patients had both, preoperative or adjuvant chemotherapy with subsequent chemotherapy for treatment of relapsed metastatic disease. None had tumors with HER2 overexpression. Of the eleven patients, 8 had tumors that expressed ERs, and 3 had triple negative carcinoma. In addition to prior chemotherapy, the eight previously treated patients whose tumors expressed ERs had previous endocrine therapy including one patient who had also CDK 4/6 inhibitor. Ten of eleven patients who had received prior chemotherapy had measurable tumor consisting in nodal, bone, soft tissue and/or visceral metastases, and one had bone metastases only. Ten patients had prior mastectomy, and one did not (Table 2). Great tumor burden was recorded in most patients. Eastern Cooperative Oncology Group (ECOG) performance status (PS) scores were 0–1, 2, 3, and 4 in eighteen, eight, six, and four patients, respectively. Four patients carried germline deleterious BRCA2 gene mutations.

## Treatment

Patients received the standard chemotherapeutic regimens comprising a combination of FUra and FA that were indicated for treatment of their disease, supplemented with pyridoxine in high doses accompanying each administration of FA plus FUra (Tables 1, and 2).

Vitamin B6 is the generic name that encompasses six interconvertible compounds (i.e., B6 vitamers), namely pyridoxine (PN); pyridoxamine (PM); pyridoxal (PL); and their respective 5′-phosphorylated forms, pyridoxine 5′-phosphate, pyridoxamine 5′-phosphate, and the cofactor pyridoxal 5′-phosphate (PLP)^33,37–40^. Pyridoxine hydrochloride, the only available marketed parenteral B6 vitamer for clinical use (in 250 mg vials) was injected iv in 30’ preceding immediately each injection of FA and then FUra for the number of days defined by the schedules of chemotherapy described below.

The dose of pyridoxine given in tandem with each FUra and FA was calculated from that determined in mouse pharmacokinetics experiments converted to human equivalent dose (HED) based on body surface area^13,37,38,41,42^. The dose of PN converted to man was 450 mg/kg, which was the greatest dose of B6 vitamer given in mice. Within the limits of the PN dose range explored in these animals^13,37^, it resulted in transient rise of intracellular PLP concentrations to the highest peak levels falling in the range of most reported Kd values for binding of PLP to apo SHMT, the requirement that supports the rationale underlying the present clinical study^13,29–32^. The first starting dose of PN accompanying each administration of FUra and FA was 1000 mg/day. Then, we practiced stepwise intra patient dose escalation of pyridoxine by increments of 500 to 1000 mg/day in subsequent courses. In absence of any form of toxicity seeming attributable to the PN recorded in prior patients, the starting daily dose of PN in next patients was increased to 2000 mg/day, and then to a maximum of 3000 mg/day corresponding in man approximately to the highest dose of pyridoxine explored in mice^13,37^, that was not exceeded in the present study. The median daily dose of PN administered to each patient resulting from dose escalation is indicated in Tables 1 and 2.

Most patients had dihydro pyrimidine dehydrogenase (DPD) phenotype assessment before initiation of treatment by measurement of plasma uracil (U) and 5,6-dihydrouracil (UH_2_) concentrations using ultra-high performance liquid chromatography tandem mass spectrometry^43^. None had uracil levels nor UH_2_/U concentration ratios indicating DPD deficiency of any degree. With the aim at improving safety, the dose of FUra given during the first course of therapy was reduced by twenty percent from protocol dose described below and was further increased to the planned dose from the second course onwards in absence of unacceptable FUra-related toxicity after the first course^44^.

### Patients with carcinomas of the digestive tract

Patients with colorectal carcinoma and with pancreas adenocarcinoma had induction treatment using the regimen said *Folfirinox* consisting in two-day courses repeated every 14 days of combined oxaliplatin (L-OHP; 85 mg/m^2^, Day 1), irinotecan (CPT11; 180 mg/m^2^, Day1), pyridoxine (PN iv in 30’ Days 1–2), folinic acid (FA; [6R,S]-5-formyl tetra hydro pteroylglutamate; [6R,S]-5-HCO-H_4_PteGlu; 200 mg/m^2^/day iv in 15’, Days 1–2), and 5-fluorouracil (FUra; 1000 mg/m^2^day, Days 1–2) distributed in one rapid iv injection (400 mg/m^2^/day), and one iv infusion during 22 h (600 mg/m^2^/day). Patients with Ras-WT colorectal carcinoma received the anti-Epidermal growth factor receptor (EGFR; HER1) chimeric monoclonal antibody cetuximab in addition to chemotherapy at 500 mg/m^2^ every 14 days. Oxaliplatin was suspended when symptoms of Grade 2 sensory peripheral neuropathy (SPN) consisting in hypoesthesia, paresthesia and/or dysesthesia lasting ≥ 1 week from the previous L-OHP injection, and/or the Lhermitte’s sign were first recorded, and then treatment was pursued with no additional change (*i.e.*, as said Folfiri regimen combined with pyridoxine). Patients with squamous-cell carcinoma of the esophagus had four-day courses repeated every 21 days of *TCbF* with combined paclitaxel (TXL; 175 mg/ m^2^, Day 1), carboplatin (CBDCA; AUC = 5 mg/ml∙min iv, Day 1), PN (iv in 30’ Days 1–4), FA (200 mg/m^2^/day, Days 1–4), and FUra (400 mg/m^2^/day iv in 2 h, Days 1–4) (Table 1). All treatment courses were accompanied by granulocyte colony-stimulating factor (G-CSF) beginning the first day of each interval between courses.

### Patients with carcinomas of the breast

Patients with breast carcinoma received four different combination regimens that were used either alone or in sequence as indicated according to each category and/or specificities of patients (Table 2). Regimens were (a) *AVCF*, consisting in 4-day courses every 21 days of doxorubicin (40 mg/m^2^ iv Day 1), vinorelbine (25 mg/m^2^ iv Day 1), cyclophosphamide (250 mg/m^2^/day iv Days 1–4), PN (iv in 30’ Days 1–4), FA, (200 mg/m^2^/day iv in 15’ Days 1–4), and FUra, (400 mg/m^2^/day iv in 2 h, Days 1–4); (b) *FAC,* consisting in 1-day courses every 21 days of doxorubicin (40 mg/m^2^ iv Day 1), cyclophosphamide (500 mg/m^2^ iv Day 1), PN (iv in 30’ Day 1), FA (200 mg/m^2^ iv in 15’ Day 1), and FUra (500 mg/m^2^ iv in 2 h); (c) *TCbF*, consisting of 4-day courses every 21 days of paclitaxel (175 mg/m^2^ iv Day 1), carboplatin (AUC = 5 mg/ml∙min iv Day 1), PN (iv in 30’ Days 1–4), FA (200 mg/m^2^/day iv in 15’ Days 1–4), and FUra (400 mg/m^2^/day iv in 2 h, Days 1–4); and (d) *VCbF* consisting of 4-day courses every 21 days of vinorelbine (25 mg/m^2^ iv Day 1), carboplatin (AUC = 5 mg/ml∙min iv Day 1), PN (iv in 30’ Days 1–4), FA (200 mg/m^2^/day iv in 15’ Days 1–4), and FUra (400 mg/m^2^/day iv in 2 h, Days 1–4). All treatment courses were accompanied by G-CSF beginning the first day of each interval between courses. Doxorubicin comprised in *AVCF* and *FAC* regimens was suspended in case of ≥ 10% decrease in left ventricular ejection fraction from baseline. Paclitaxel included in the *TCbF* regimen was suspended when symptoms of sensory peripheral neuropathy consisting in hypoesthesia, paresthesia and/or dysesthesia of any intensity, and/or limb pain were first recorded. Vinorelbine-containing chemotherapy (i.e., *VCbF*) was indicated for patients who had severe hematopoietic impairment or prior taxane-induced toxicity and was used in substitution for *TCbF* in cases of paclitaxel-induced SPN occurring during the study. Of eighteen patients who were not previously treated and whose tumors did not overexpress HER2, thirteen received an initial sequence of anthracycline-containing chemotherapy (4–6 courses), and then a succession of *TCbF* courses followed by *VCbF* in substitution to *TCbF* when needed for the reasons above. Anthracycline-containing chemotherapy was *AVCF* in eleven patients and *FAC* in 2. *AVCF* and *FAC* were avoided in five patients owing to hematologic impairment in two, to cardiorespiratory dysfunction in two patients, and to prior anthracycline-containing therapy in one patient; these 5 patients received taxane- and vinorelbine-containing regimens only. The seven patients who had not been previously treated, and whose tumors overexpressed HER2 (3 +) received a succession of *TCbF* courses combined with the anti HER2/neu humanized monoclonal antibodies trastuzumab (6 mg/kg iv every 21 days), and pertuzumab (420 mg/patient iv every 21 days). *TCbF* courses were followed by *VCbF* in substitution to *TCbF* when needed for the reasons above. Induction chemotherapy for the eleven patients who had been previously treated consisted in *TCbF* in eight patients, *VCbF* in two patients who had received prior paclitaxel, and one patient that had not received prior anthracycline-containing chemotherapy received *AVCF* followed by *VCbF* (Table 2). Premenopausal patients, whose tumors expressed ERs, received long-term luteinizing hormone-releasing hormone analog (LHRHa).

#### Duration of induction treatment and post-induction care given to patients

Patients with digestive tract and breast carcinomas had induction treatment courses repeated until antitumor response of the estimated maximum degree was attained, and then chemotherapy was either discontinued or pursued with an a priori undefined but limited number of courses in a personalized way according to patient’s condition and decisions from patients, referring oncologists, and clinical meetings. Patients with breast carcinoma who had completed induction chemotherapy whose tumors expressed ERs received long-term aromatase inhibitor therapy accompanied with LHRHa in premenopausal patients, and those whose tumor overexpressed HER2 continued with three-weekly trastuzumab during one supplementary year.

#### Objectives of the study and assessment of antitumor activity

The primary objective of the study was response rate and magnitude of response. Antitumor response was assessed by studying variation of the sum of diameters of anatomically measurable tumors according to RECIST (Response Evaluation Criteria in Solid Tumors)^45^, and that of peak standard ^18^FDG uptake value normalized by lean body mass (SULpeak) of targets according to PERCIST (Positron Emission Tomography (PET) Response Evaluation Criteria in Solid Tumors)^46^, together with periodic clinical examination including follow-up of non-measurable tumor involvement, and measurement of plasma tumor markers. Rapidity of tumor shrinkage was the time from start of therapy to first observation of objective antitumor response.

Event-free survival (EFS) was a secondary objective owing to the varied types of carcinomas of diverse phenotypic characteristics in patients whose single common thread was to have been treated with induction regimens including FUra, folinic acid and high dose pyridoxine in tandem. EFS was the time from initiation of induction treatment to the first tumor progression event or to the time of death of any cause unrelated to carcinoma progression.

In addition to clinical and metabolic assessment, antitumor potency of treatment was evaluated by histopathologic assessment of residual tumor performed at variable times after induction therapy in selected patients who had attained either a complete or a partial response of great magnitude. Resection of various types depending on the site of primary and local and metastatic extension was performed in patients with tumor developed in one anatomic area without detectable metastasis or with metastasis in limited numbers that had disappeared under induction chemotherapy, or that had been reduced under induction chemotherapy to be accessible to surgical eradication intent. Histopathologic assessment was also obtained by imaging-guided biopsy in stage IV patients who had persistent focal residual images of unknown significance after induction treatment in sites of prior tumor involvement, or by trephine bone biopsy in patients with initial extensive osteomedullary involvement.

#### Assessment of toxicity

Assessment was performed before initiation of each cycle of therapy according to Common Terminology Criteria for Adverse Events (CTCAE) v5. Particular attention was put on assessment of sensory peripheral neuropathy with emphasis to symptoms associated with the neurotoxic drugs used in the studies as described above, because peripheral nerve toxicity was reported ensuing intake of pyridoxine in very high doses for extended periods of time^47,48^.

#### Statistical and survival analysis

Analyses were performed with R software version 4.2.1. Univariate survival models were fitted with *Survival* R-package version 3.5–7. Kaplan–Meier plots and 95% confidence intervals were drawn with *Survminer* R-package version 0.4.9.

## Results

Of fifty patients included, forty-seven responded to therapy (response rate, 94%) including 12 patients with carcinomas of the digestive tract, and 35 with breast carcinoma, and three patients had no change or progressive disease. Induction treatment including FUra, FA, and PN in tandem resulted in antitumor responses of early onset and great magnitude. Antitumor response was assessed according to RECIST in forty-nine patients, and to PERCIST in forty-one**.** Forty patients were assessed according to both methods. Twenty-five responders underwent pathologic assessment of response (Tables 1, and 2).

## Patients with carcinomas of the digestive tract

### Patients with colorectal adenocarcinoma

**Figure 2:**
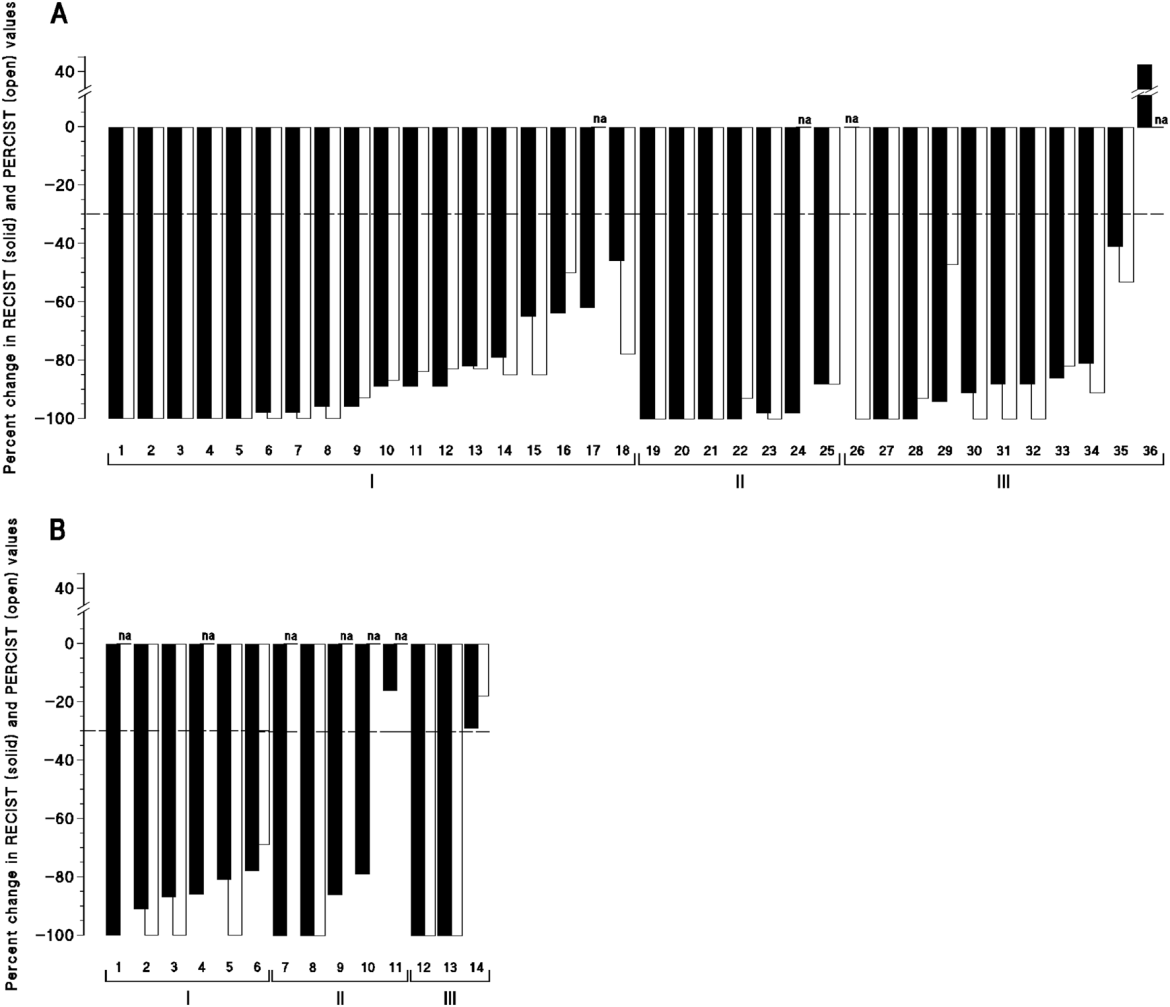
Magnitude of clinical and metabolic antitumor response in advanced digestive tract, and breast carcinoma patients in advanced stages treated with regimens comprising FUra, folinic acid, and pyridoxine in tandem. Patients with breast carcinoma (**A**) are represented in three groups comprising I, previously untreated patients whose tumors did not overexpress HER2 (1–18); II, previously untreated patients whose tumors overexpressed (3 +) HER2 (19–25); and III, previously treated patients whose tumors did not overexpress HER2 (26–36). Patients with previously untreated digestive tract carcinomas (**B**) are represented in three groups which comprise I, patients with colorectal carcinoma (1–6); II, patients with carcinoma of the pancreas (7–11); and III, patients with squamous-cell carcinoma of esophagus (12–14). Patients are numbered in the same order as in Tables 1, and 2, and Fig. 3. In patients who had great numbers of targets who attained a partial response accompanied by disappearance of most metastases, calculations of percent reduction in sum of diameters (RECIST; Response Evaluation Criteria in Solid Tumors) were done by size comparison of remaining images at the time of assessment with these same tumor images present before treatment. Metabolic response was assessed by the percent variation in peak standard ^18^FDG uptake value normalized by lean body mass (SUL_peak_) obtained by PET scan (PERCIST; Positron Emission Tomography (PET) Response Evaluation Criteria in Solid Tumors). Thirty-nine responders were assessed by both methods, and eight did not. The discontinuous line at − 30%, represents the limit between no change and antitumor response. Percent change from baseline according to RECIST, and PERCIST are represented with solid bars, and open bars, respectively. Na, not applicable.

**Figure 3:**
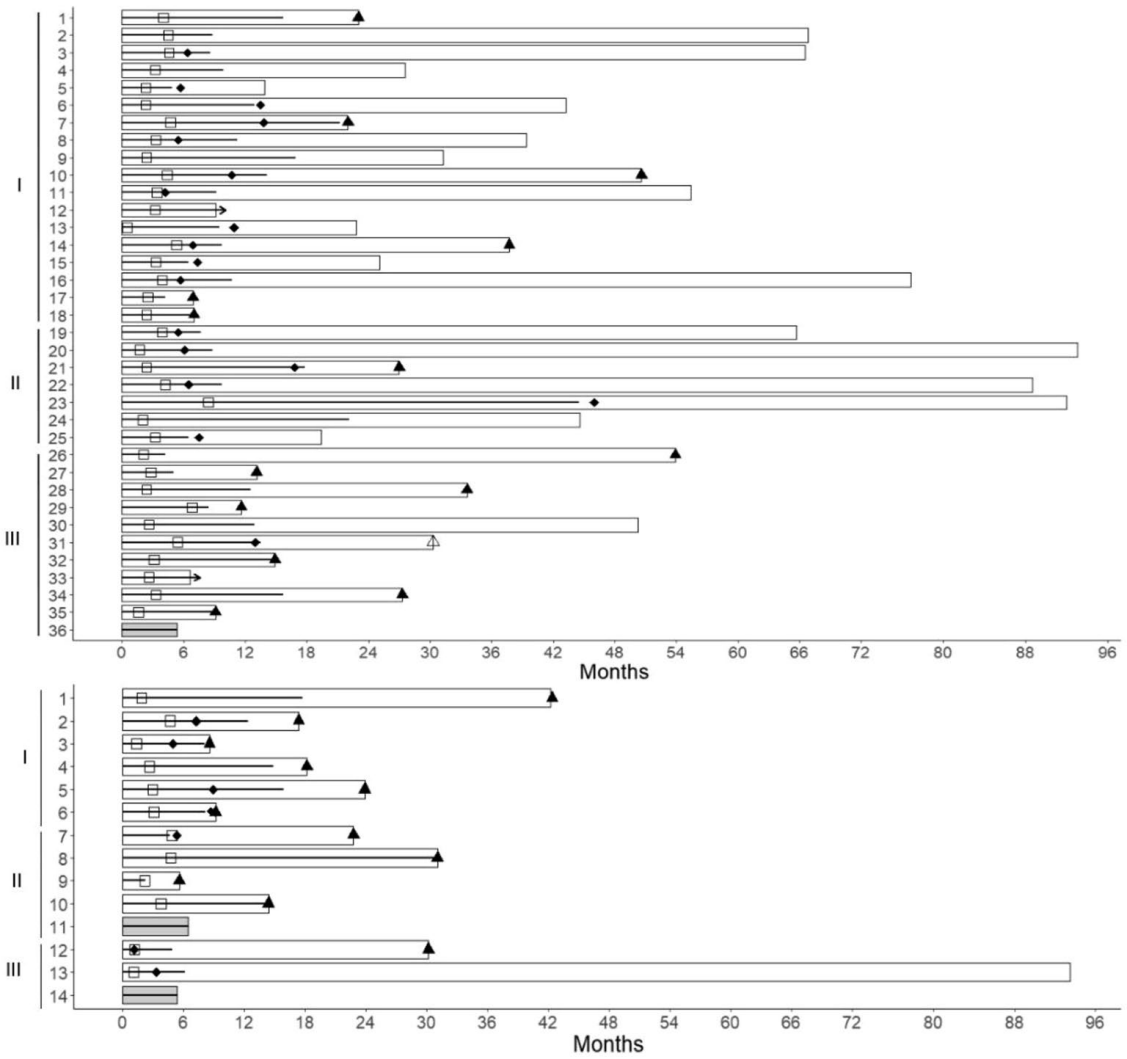
Chronological sequence of events in patients with advanced digestive tract and breast carcinoma in advanced stages treated with regimens including FUra, folinic acid and pyridoxine in tandem. Groups of patients with breast cancer (top) comprise 1, previously untreated patients whose tumors did not overexpress HER2 (1–18); II, previously untreated patients whose tumors overexpressed (3 +) HER2 (19–25); and III, patients who had received prior chemotherapy whose tumors did not overexpress HER2 (26–36). Groups of previously untreated patients with digestive tract neoplasms (bottom) comprise I, colorectal carcinoma patients (1–6); patients with carcinoma of the pancreas (7–11); and patients with squamous-cell carcinoma of esophagus (12–14). Patients are numbered in the same order as in Tables 1, and 2, and Fig. 2. Open bars represent event-free survival times. EFS times refer to progression-free survival in all patients except for one who had fatal disease without breast carcinoma progression. Dark grey bars represent time required to final evaluation in the three patients that did not respond to therapy. Bold black lines within bars represent duration of treatment comprising FUra, folinic acid and pyridoxine in tandem, and arrow indicates ongoing treatment at the time of present evaluation. Open squares indicate the time when a response to therapy was first recorded, *i.e.*, a reduction in sum of diameters and/or SUL_peak_ value by > 30%. Solid diamonds represent the time when pathologic assessment was performed. Solid triangles indicate the time when tumor progression was recorded in prior responders to therapy. Open triangle indicates time of event leading to withdrawal from study in a patient with persisting response to therapy.

**Figure 4:**
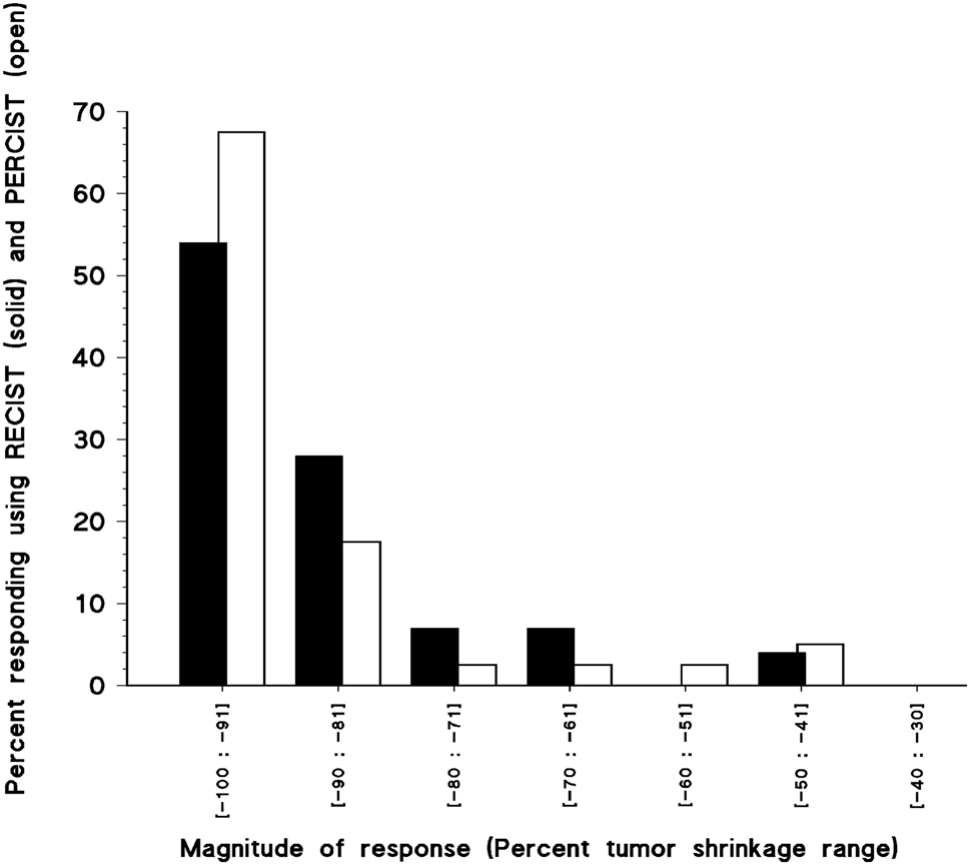
Magnitude of antitumor response in percent tumor shrinkage range in forty-seven patients with advanced digestive tract and breast carcinomas who responded to regimens including FUra, folinic acid, and pyridoxine in tandem. Distribution of antitumor responses according to their magnitude was determined in forty-six patients who were assessed clinically according to RECIST (solid bars), and forty patients who were assessed by PET scan according to PERCIST (open bars). Of the twenty-five patients who attained antitumor responses of equal or greater than 91 percent tumor reduction, sixteen achieved complete responses.

Of six patients, one attained a CR and 5 had clinical PRs with percent tumor reduction of 91, 87, 86, 81, and 78%, accompanied with disappearance of most metastases (Table 1; Figs. 2, 3, and 4). Two partial responders had no residual tumor assessed by resection surgery in one, and endoscopy-guided biopsy in the second. EFS times were 9, 9, 17, 18, 24, and 42 months.

### Patients with pancreas adenocarcinoma

Of the five patients, two attained a CR, two had a PR with percent reduction in sum of diameters of 86, and 79%, and one patient had no change. One complete responder had pancreaticoduodenectomy resulting in removal of residual tumor staged ypT3N0M0. EFS times were 6, 14, 23, and 31 months.

### Patients with squamous‑cell carcinoma of the esophagus

Two patients attained CRs, and one had no change. One complete responder had endoscopic ultrasound-guided biopsy of primary and nodes that did not find residual tumor. The second complete responder had esophagectomy with no residual tumor in resected tissue. EFS times were 30, and 94 + months.

Of the fourteen patients, eight including 7 responders had both clinical and PET scan assessment (Table 1 and Fig. 2; of these, six attained metabolic CRs, and one patient had metabolic PR with reduction of SULpeak by 69%. Clinical and PET scan assessment was in conformity in three patients who achieved clinical CRs; discrepancies between both methods by 9–19% (mean difference, 13%) were observed in 4 partial responders. Times to attain a response ranged from 1.1 to 4.9 months (median, 2.8 months).

## Patients with breast carcinoma

### Patients without prior chemotherapy whose tumors did not overexpress HER2

Of eighteen patients, 5 attained clinical CRs and 13 had PRs with percent reduction in sum of diameters by 98, 98, 96, 96, 89, 89, 89, 82, 79, 65, 64, 62, and 46%, accompanied by disappearance of metastases in 12 patients out of 14 with stage IV carcinoma (Table 2; Figs. 2, 3 and 4). Of seventeen patients who were assessed by PET scan, 8 attained metabolic CRs and 9 patients had metabolic PRs with percent reduction of SULpeak value by 93, 87, 85, 85, 84, 83, 83, 78, and 50%. Of six patients who underwent mastectomy, one had pCR (*ypT0N0*), and five had residual tumor staged *ypT0N1(mi)*, *ypT1bN0*, *ypT1bN1a*, *ypT1cN1a*, and *ypT3N1a*. Two patients with prior mastectomy who had surgical resection of previously involved areas had small amounts of residual tumor < 1 cm in diameter in resected tissue. Of three patients assessed by percutaneous biopsy, one had no residual breast tumor found, and two had persistent carcinoma. Of the 18 patients, twelve did not have tumor progression after EFS times of 9 + 14 + , 23 + , 25 + , 28 + , 31 + , 39 + , 43 + , 55 + , 67 + , 67 + , and 77 + months, and the other six patients had tumor growth after EFS times of 7, 7, 22, 23, 38, and 51 months (Table 2, and Fig. 3).

### Patients without prior chemotherapy whose tumors overexpressed HER2

Of seven patients, four attained clinical CRs, and 3 had PRs with reduction in sum of diameters of 98% in two patients, and of 88% in one; responses were accompanied by disappearance of metastases in the four stage IV patients. Of six patients who were assessed by PET scan, 4 attained metabolic CRs and two had metabolic PRs with reduction of SULpeak value by 93, and 88%. Of five patients who underwent mastectomy, pathologic CRs (*ypT0N0*) were achieved by 3 patients, one had minimal residual tumor staged *ypT1aN1(mi),* and one patient had residual invasive and intraductal primary staged *ypT1bN0* that did not overexpress HER2 anymore. The single patient assessed by percutaneous biopsy did not have residual tumor found. Of the seven patients, six did not have tumor progression after PFS times of 19 + , 45 + , 66 + , 89 + , 92 + , and 93 + months, and one had tumor growth after EFS time of 27 months (Table 2, and Fig. 3).

### Patients who had received prior chemotherapy

Ten patients responded to therapy, and one had progressive disease. Of nine responders with measurable tumor, 2 attained clinical CRs, and 7 had PRs with reduction in sum of diameters by 94, 91, 88, 88, 86, 81, and 41%. One patient with non-measurable tumor who had exclusive bone metastases experienced metabolic CR. Responses were accompanied by disappearance of most metastases. Of ten patients who were evaluated by PET scan, 5 attained metabolic CRs and 5 had PRs with SUL_peak_ reduction by 93, 91, 82, 53, and 47%. One partial responder had no residual tumor as assessed by percutaneous biopsy. Of the ten responders, two patients did not have tumor progression after EFS times of 7 + , and 50 + months, seven had tumor progression after EFS times of 9, 12, 13, 15, 27, 34, and 54 months from start of therapy, and one patient with prior genotoxic chemotherapy and radiotherapy had fatal AML diagnosed 18 months after completion of treatment without carcinoma progression; EFS time was 30 months (Table 2, and Fig. 3). Of the eleven patients, nine had received FUra as part of their previous regimens of chemotherapy, including five who had FUra and folinic acid.

Of thirty-five responders with breast carcinoma, 32 had clinical evaluation of response together with PET scan assessment. Conformity in percent reduction as assessed by both methods was found in 10 patients including 9 who attained clinical CRs; moderate disparity in magnitude of response by < 15% reduction rate (mean difference in percent reduction, 6%) was found in 19 patients; and disparity by 20, 32, and 47 per cent reduction was recorded in 3 patients. Times to attain a response ranged from 0.5 to 6.8 months (median, 3.2 months).

## Response rate, magnitude of response, and rapidity of tumor shrinkage

Forty-seven patients responded to therapy (94%), including 16 complete responders (CR rate, 32%). Overall, the 47 responders had median magnitude of response of -93% according to RECIST (mean, − 89%) and of − 100% (mean, − 91%) according to PERCIST (Tables 1, and 2; Figs. 2, 3 and 4). Twenty-five patients (50%) attained clinical responses of ≥ 91% tumor reduction. Of these, twenty-three patients who were also assessed by PET scan had SUL_peak_ reduction by > 91% in 22 patients, and reduction below 90% in one. Median magnitude of response of the twelve responding patients with digestive tract carcinomas according to RECIST was − 89% (mean, − 91%; range, − 78 to − 100%), and it was − 100% (mean, − 96%; range, − 69 to − 100%) according to PERCIST in seven responders who were assessed by PET scan. Median magnitude of antitumor response of the thirty-four responders with breast carcinoma who were assessed according to RECIST was − 95% (mean, − 88%; range, − 41 to − 100%), and it was − 100% (mean, − 90%; range, − 47 to − 100%) according to PERCIST in 33 who were assessed by PET scan. Median time to attain a response was 2.8 months, and 3.2 months in patients with digestive tract, and breast carcinomas, respectively. Responders who had bone metastases experienced osseous remineralization, and serosal effusion disappeared in patients who had pleura and/or pericardium involvement. Objective responses were accompanied by decrease in plasma tumor marker levels in all patients who had elevated markers before start of treatment (Tables 1, and 2).

## Histopathologic assessment of antitumor response

Overall, of 25 patients who underwent pathologic assessment, eleven did not have residual tumor found, seven had minimal residual disease within resected tissue whose total diameter was ≤ 10 mm, and seven had either residual tumor of greater than 10 mm in diameter in resected tissue, or persistence of metastatic tumor of any dimension in viscera, bone, or skin (Tables 1, and 2).

## Event-free survival

**Figure 5:**
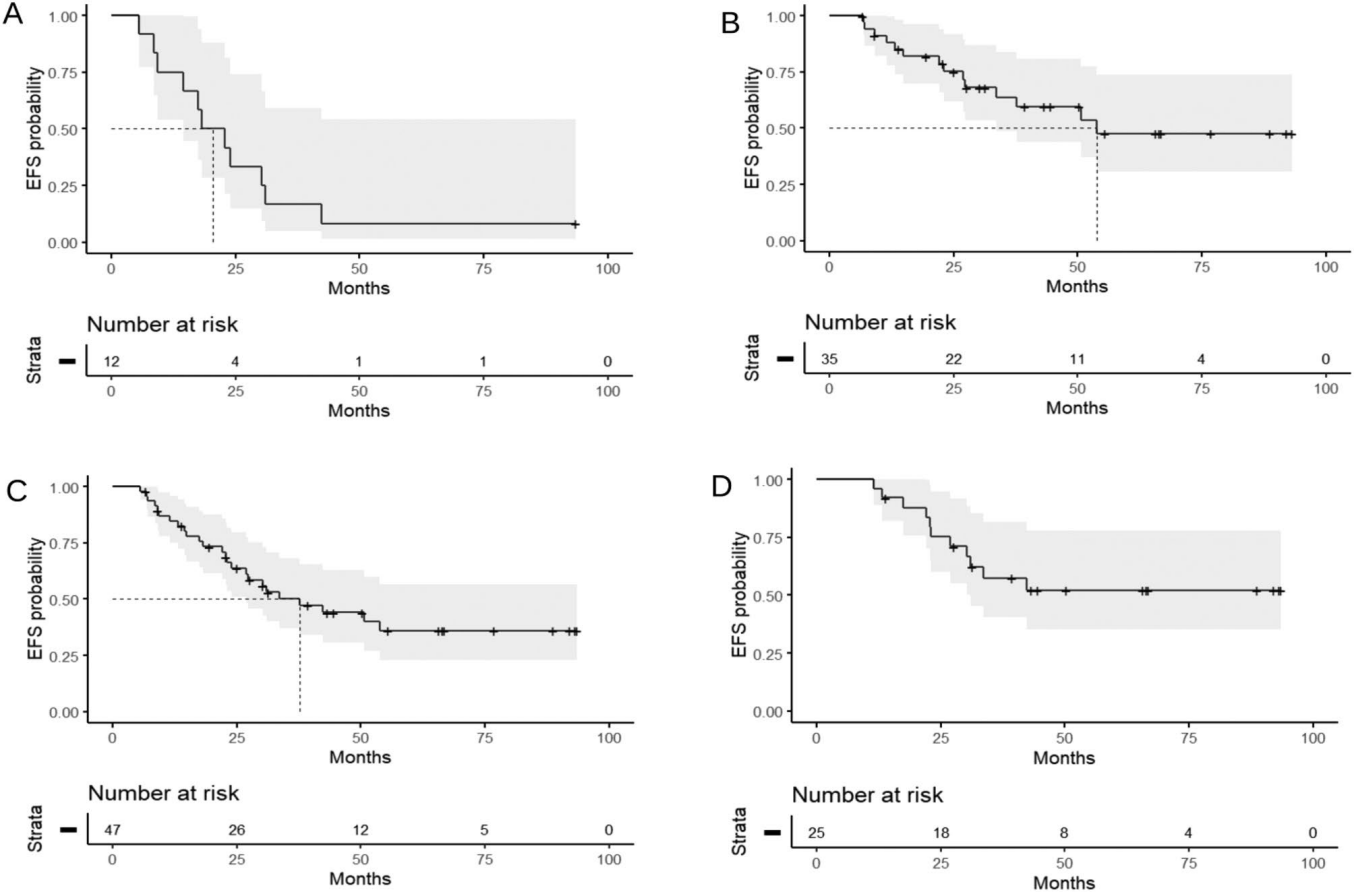
Event-free survival (EFS) probability of patients with digestive tract and breast carcinomas in advanced stages treated with regimens including FUra, folinic acid, and pyridoxine in tandem. Event-free survival (EFS) probability of 12 responders with digestive tract carcinomas (**A**), 35 responders with breast carcinoma (**B**), 47 responders with both, digestive tract, and breast carcinomas (**C**), and 25 patients with both, digestive tract, and breast carcinoma who attained clinical responses ≥ 91 percent tumor reduction (**D**). EFS times refer to progression-free survival in all patients except for one patient with breast carcinoma who had fatal disease without carcinoma progression. Dotted lines represent median EFS time, and shadowed areas represent 95% confidence interval (CI). Median EFS values were A, 20.5 months (CI, 0.28–0.88); B, 53.9 months (CI, 0.31–0.74); C, 37.7 months (CI, 0.34–0.66); and D, median not reached, patients having a probability of being event-free of 52% (CI, 0.35–0.78) from 42.4 months.

Event-free survival (EFS) times of the forty-seven responders refer to progression-free data, except for one patient with breast carcinoma who had fatal disease in absence of carcinoma progression (Tables 1, and 2; Figs. 3, and 5). Median EFS time of the 12 responders with digestive tract carcinomas was 20.5 months (95% CI, 0.28–0.88), and median EFS time of the 35 responders with breast carcinoma was 53.9 months (95% CI, 0.31–0.74). The 47 responders in the two studies had median EFS time of 37.7 months (95% CI, 0.34–0.66). Median EFS time was not reached in the group of 25 patients who had tumor shrinkage ≥ 91% reduction; these patients had a probability of remaining event-free of 52% (95% CI, 0.35–0.78) from 42.4 months onwards.

## Toxicity

Toxicity of unusual form or greater degree than that expected with each regimen of chemotherapy in absence of high dose B6 vitamer was not recorded. No dose reductions of any cytostatic agent nor increasing intervals between courses due to toxic events were required. Overall, the fifty patients received 841 courses of chemotherapy (median number per patient, 14 courses). Median PN dose administered to the fifty patients was 1000 mg/day, 1500 mg/day, 2000 mg/day, 2500 mg/day and 3000 mg/day in 15, 3, 9, 1, and 22 patients, respectively (Tables 1 and 2). Neurologic toxicity occurred only in patients who were treated with regimens including oxaliplatin (*Folfirinox*) or paclitaxel (*TCbF*), SPN events having the clinical characteristics of neuropathy caused by each of these drugs. All eleven patients with digestive tract carcinomas who received l-OHP as part of the *Folfirinox* had this drug suspended when Grade 2 SPN was recorded. Interruption of l-OHP occurred after patients had received cumulative total doses from 442 to 1044 mg/m^2^ (median, 713 mg/m^2^). Thirty-three patients with breast carcinoma received one to 23 courses (mean, 7 courses) of the regimen *TCbF* that was suspended when symptoms of SPN were recorded. Nineteen patients did not experience clinical SPN after having received paclitaxel from 150 to 1240 mg/m^2^ (mean, 860 mg/m^2^), and fourteen did; the latter received paclitaxel from 156 to 2937 mg/m^2^ (mean, 1435 mg/m^2^). Of these, ten patients had Grade 1 SPN, and four had Grade 2, including one patient with acute limb pain and severe paresthesia occurring after a single course of therapy. All patients experienced progressive improvement of clinical signs after discontinuation of oxaliplatin or paclitaxel, and no aggravation was noted in any despite continuation of FUra, FA, and PN in tandem, including patients who received vinorelbine as part of the regimen *VCbF* after suspension of the *TCbF*.

## Discussion

Ninety-four percent of patients with carcinomas in advanced stages with poor clinical prognosis responded to regimens including FUra, FA, and PN in tandem with median tumor reduction of − 93% according to RECIST, and of -100% according to PERCIST, including 25 patients (50%) who attained responses of ≥ 91% reduction of whom 16 (32%) achieved a complete response. Moreover, of 25 responders that underwent pathologic assessment, eleven (44%) did not have tumor found, and 7 (28%) had minimal residual tumor. Median times required to attain antitumor responses were brief, of 2.8 months, and 3.2 months for patients with digestive tract, and breast carcinomas, respectively. Event-free survival probability of the 25 patients who attained responses ≥ 91% tumor reduction was 52% from 42.4 months onwards which indicate the importance at achieving antitumor responses of great magnitude for improvement of long-term outcome of patients with advanced stage carcinomas.

Response rates and magnitude of responses reported herein are remarkably superior to that reported elsewhere for patients with similar types of carcinomas in advanced stages, which suggest that addition of vitamin B6 in high dose strongly enhances the antitumor activity of combination regimens comprising FUra and FA. Currently used standard combination chemotherapy regimens produce effective but limited antitumor effect in advanced breast carcinoma patients whose response rates range between 40 and 60%, with 10% to 15% CR rate with no recognized standard regimen, since taxane-containing combinations were only modestly better than anthracycline based regimens for response rate and PFS^49,50^. Platinum derivative-containing regimens were slightly more potent than non-platinum treatments, this difference being more marked in the subset of patients with triple negativebreast carcinoma^51^. FUra and folinic acid as single agents or in combination with other cytostatics for patients with advanced breast carcinoma led to response rates ranging from 39 to 72%, with 12% mean overall CR rate^37^. Treatment with *Folfirinox* yielded antitumor responses in 75%-85% of patients with advanced colorectal adenocarcinoma^52,53^, and *Folfox* (a combination of FUra, FA, and l-OHP) together with the anti EGFR monoclonal antibody panitumumab produced responses in approximately 70% of Ras-WT patients of whom about 35% had response depth of ≥ 71% tumor reduction^54^. Complete response rates ranging 0–8% were reported in these studies^52–54^. Approximately 30% of patients with pancreas carcinoma attained antitumor responses with *Folfirinox*^55^. *Nalirifox*, a *Folfirinox*-related regimen, and combined nanoparticle albumin-bound (Nab) paclitaxel with gemcitabine yielded response rates in 42%, and 34–36% of patients, respectively with CR rates ≤ 1%^56,57^.

We did not find hints for additional toxicity with reference to that commonly reported with standard regimens of chemotherapy in absence of pyridoxine. Occurrence of oxaliplatin- and paclitaxel-induced SPN did not differ in incidence, grade, and cumulative doses from that previously reported with these agents as scheduled in standard regimens^58,59^. However, definitive conclusions require comparative studies designed for specific assessment of toxicity.

Diverse methods aiming at clinical improvement of the effect of FUra and FA including use of the pure [6S]-diastereoisomer of folinic acid or introduction of changes in doses and modalities of administration of the compounds did not convincingly succeed^44,60,61^. In recent years, we attempted at diverting pharmacologically the folate interconversion network to expand 5,10-CH_2_-H_4_PteGlu pools in cancer cells. Improvement of FUra cytotoxicity accompanied with decrease in free TS levels resulting from enzymatic elimination of L-methionine was found in vitro, but this approach could not find clinical application yet^22^. In the present study we translated in the clinics a method that facilitates folate conversion by stimulating the PLP-dependent SHMT catalyzed synthesis of CH_2_-H_4_PteGlu from H_4_PteGlu^13^.

Further Phase II, and Phase III clinical trials including FUra, folates and vitamer B6 in tandem are urgently needed. Confirmation of results reported herein would offer the possibility for additional improvement of regimens comprising FUra, folates, and B6 vitamers in tandem. Theory and experimental data indicate that increase of the cytotoxic activity of FUra depends to a still undetermined limit on the level of intracellular expansion of PLP pools to enhance the SHMT-catalyzed synthesis of CH_2_-H_4_PteGlu within cancer cells exposed to FdUMP and reduced folate in a time range permitting sustained inhibition of the TS through stabilization of the ternary complex [TS-FdUMP-5,10-CH_2_-H_4_PteGlu] by enhancement of CH_2_-H_4_PteGlu mass flux. Studies should determine the concentration of PLP over time to be achieved for maximum potentiation of the fluoropyrimidines which, theoretically, is the amount eventually up to several folds the K_d_ value for binding of cofactor required for SHMT active site occupation rate needed to attain the highest activity of the enzyme^13,31,36^. To reach this goal, studies should first explore the appropriate dose and type of B6 vitamer for optimum intracellular PLP pool expansion to be combined with FUra, and folates. The synergistic cytotoxic potentiation was obtained in vitro by cell exposure to FUra, FA, and high concentration PLP, but the intracellular cofactor levels attained under this experimental condition were not measured^13^. Yet, cell uptake of PLP was reported to be limited, resulting in intracellular concentrations below 10% that in supernatant^62,63^. Moreover, increase of intracellular PLP in mouse up to levels within the range of most reported K_d_ for binding of cofactor to apo SHMT were attained after parenteral administration of PN, and PM at the greatest dose tested of 450 mg/kg, i.e., approximately the equivalent in man of 3000 mg corresponding to the highest dose of PN accompanying each FUra and folinic acid used in the present clinical study^13,37^. Furthermore, the intracellular PLP and PMP peak concentration levels, and area under the concentration versus time curves (AUCs) in mice that received PM were much greater than that in animals having received PN^13,37^. The disparity of intracellular pharmacokinetics between these unphosphorylated B6 vitamers lies in marked differences for their respective enzymatic conversion in cofactor, and catabolism. Both PM and PN are phosphorylated by pyridoxal kinase in PMP and PNP, and then oxidized to PLP by pyridoxine 5′-phosphate oxidase, whose affinity for PMP is greater than for PNP. In addition, PMP is converted to PLP through a pyridoxamine aminotransferase^39,40,64,65^. Furthermore, the specificity constant (V_max_/K_m_) of the 5′-pyridoxal phosphate phosphatase, the enzyme that catabolizes intracellular phosphorylated B6 vitamers, is lowest for PMP^13,37,66^. These data indicate that parenteral PM may possess an advantage over PN to expand intracellular PLP pools.

## Data Availability

We state that all data generated during this study are included in the article. Data and materials are reported in the text under Methods, and Results sections, in Tables 1 and 2, and Figs. 2, 3, 4, 5. Supplementary iconographic data for all the patients are available on demand to Corresponding author.
